# Combination therapy of bevacizumab and galunisertib extends TVN time window

**DOI:** 10.1016/j.omton.2024.200888

**Published:** 2024-10-15

**Authors:** Ling Yin

**Affiliations:** 1 College of Medicine, University of Florida, Gainesville, FL, USA

## Main text

Tumor vascular normalization (TVN) enhances tumor perfusion and reduces metastasis, making it a promising alternative strategy for anti-angiogenic cancer treatment.^1^ The vascular endothelial growth factor (VEGF) family, known for its significant pro-angiogenic activity and anti-apoptotic effects on endothelial cells, consists of several members, including VEGF-A, VEGF-B, VEGF-C, VEGF-D, VEGF-E, VEGF-F, PlGF, and EG-VEGF.^2^ Transforming growth factor β (TGF-β) regulates angiogenesis, acting as a pro-angiogenic factor at low concentrations and an anti-angiogenic factor at high concentrations.^3^ Both VEGF and TGF-β play crucial roles in tumor proliferation, angiogenesis, growth, migration, and invasion.

In a recent article published in *Molecular Therapy Oncology* titled “Galunisertib promotes bevacizumab-induced vascular normalization in nasopharyngeal carcinoma: multi-parameter MRI,” the authors highlight the role of the TVN time window in restoring the integrity of tumor blood vessels, enhancing tumor radiosensitivity, and improving the efficacy of anti-cancer therapies. This is essential for cancer combination therapies, preclinical studies, clinical trials, and immunotherapy drug discovery. To address the limitations of the short-term TVN time window in clinical applications for nasopharyngeal carcinoma (NPC), the authors propose a combined treatment strategy using the VEGF monoclonal antibody bevacizumab and the TGF-β receptor type I kinase inhibitor galunisertib. This combination extends the TVN time window, broadening the clinical application of TVN in anti-angiogenesis cancer therapy.

Several factors contribute to the extension of the TVN time window with combined targeted therapy. First, inhibiting HIF-1α expression improves the tumor’s hypoxic microenvironment. Second, simultaneously targeting the VEGF/VEGF-R axis and the TGF-β pathway helps regulate the balance between pro-angiogenic and anti-angiogenic factors. Third, reversing the EMT processes in angiogenesis decreases the expression of angiogenic factors (such as E-cadherin, vimentin, and N-cadherin) and LAMC2 via the JNK/AP1 pathway. Compared to monotherapies with either bevacizumab or galunisertib, the combination therapy demonstrates better antitumor effects, including smaller tumor size, greater anti-proliferative effects, reduced tumor microvascular density, and improved vascular normalization markers like the pericyte coverage index and collagen IV. It also shows lower toxicity, maintaining the integrity of tissue structures in the liver, kidneys, and spleen in an NPC patient-derived xenograft (PDX) mouse model. Most notably, the combined therapy extends the TVN time window from day 3 to at least day 25 post-treatment, compared to a TVN time window of day 3 to 14 with single-agent treatments.

Multiparametric magnetic resonance imaging (MRI), including dynamic contrast-enhanced MRI (DCE-MRI), intravoxel incoherent motion diffusion-weighted imaging (IVIM-DWI), and amide proton transfer (APT) imaging, has been used to investigate normal and pathological tissues in cancer research.^4^^,^^5^ The authors conducted DCE, IVIM, and APT-MRI at different time points, measuring parameters such as Ktrans, f, and D∗ to monitor TVN efficacy and the tumor’s response to therapy. Their findings suggest that multiparametric MRI can replace pathological indicators for noninvasive and real-time monitoring, allowing for early and accurate assessment of tumor responses to anti-angiogenesis therapy and the TVN time window, which could aid in the clinical application of TVN in anti-angiogenesis cancer treatment.

Despite these advances, there are still areas requiring further research, such as the molecular mechanisms by which LAMC2 regulates tumor angiogenesis and vascular normalization, as well as the advantages of using multiparametric MRI to monitor the TVN process.

## Declaration of interests

The author declares no competing interests.
